# SB203580 Modulates p38 MAPK Signaling and Dengue Virus-Induced Liver Injury by Reducing MAPKAPK2, HSP27, and ATF2 Phosphorylation

**DOI:** 10.1371/journal.pone.0149486

**Published:** 2016-02-22

**Authors:** Gopinathan Pillai Sreekanth, Aporn Chuncharunee, Aunchalee Sirimontaporn, Jutatip Panaampon, Sansanee Noisakran, Pa-thai Yenchitsomanus, Thawornchai Limjindaporn

**Affiliations:** 1 Department of Anatomy, Faculty of Medicine Siriraj Hospital, Mahidol University, Bangkok, Thailand; 2 Medical Biotechnology Unit, National Center for Genetic Engineering and Biotechnology, National Science and Technology Development Agency, Bangkok, Thailand; 3 Division of Molecular Medicine, Department of Research and Development, Faculty of Medicine Siriraj Hospital, Mahidol University, Bangkok, Thailand; Institute of Biochemistry and Biotechnology, TAIWAN

## Abstract

*Dengue virus* (DENV) infection causes organ injuries, and the liver is one of the most important sites of DENV infection, where viral replication generates a high viral load. The molecular mechanism of DENV-induced liver injury is still under investigation. The mitogen activated protein kinases (MAPKs), including p38 MAPK, have roles in the hepatic cell apoptosis induced by DENV. However, the *in vivo* role of p38 MAPK in DENV-induced liver injury is not fully understood. In this study, we investigated the role of SB203580, a p38 MAPK inhibitor, in a mouse model of DENV infection. Both the hematological parameters, leucopenia and thrombocytopenia, were improved by SB203580 treatment and liver transaminases and histopathology were also improved. We used a real-time PCR microarray to profile the expression of apoptosis-related genes. Tumor necrosis factor α, caspase 9, caspase 8, and caspase 3 proteins were significantly lower in the SB203580-treated DENV-infected mice than that in the infected control mice. Increased expressions of cytokines including TNF-α, IL-6 and IL-10, and chemokines including RANTES and IP-10 in DENV infection were reduced by SB203580 treatment. DENV infection induced the phosphorylation of p38MAPK, and its downstream signals including MAPKAPK2, HSP27 and ATF-2. SB203580 treatment did not decrease the phosphorylation of p38 MAPK, but it significantly reduced the phosphorylation of MAPKAPK2, HSP27, and ATF2. Therefore, SB203580 modulates the downstream signals to p38 MAPK and reduces DENV-induced liver injury.

## Introduction

*Dengue virus* (DENV) infection is one of the most important mosquito-borne viral diseases with high incidence in tropical and subtropical regions. The clinical signs of DENV infection reflect the different levels of severity including dengue fever or dengue hemorrhagic fever, or dengue shock syndrome (DSS). Patients with more severe forms of the disease display hemorrhagic disorders, including plasma leakage, thrombocytopenia, hemoconcentration, and multi-organ failure [1–6]. Liver transaminase (alanine transaminase [ALT] and aspartate transaminase [AST]) levels increase in both DENV-infected patients [7–10] and murine models of DENV infection [11–15].

Hepatic cell apoptosis, which is related to the pathogenesis of DENV infection, has been observed both *in vitro* and *in vivo* [16–18]. DENV infection contributes to apoptosis by inducing the expression of cytokine TRAIL, observed in the hepatic cell line, HepG2 [19]. DENV infection with increased cytokine expression can proceed to liver injury. The expression of tumor necrosis factor α (TNF-α), one of the predominant pro-inflammatory cytokines, is increased in DENV infection [20–25]. The Fas receptor (FasR) is the member of the TNF death receptor family and its signaling also contributes to DENV-mediated apoptosis [26, 27]. Furthermore, DENV infection causes mitochondrial dysfunction, which contributes to hepatic cell injury [28, 29]. Activation of caspase 9 and caspase 3 is seen in DENV-infected human umbilical vascular endothelial cells (HUVECs) suggesting the involvement of mitochondrial caspase and the intrinsic pathway of apoptosis [30]. The involvement of intrinsic pathway in DENV infection is also reported in other cell types [31, 32]. Therefore, DENV infection induces both extrinsic and intrinsic pathways of apoptosis.

Mitogen-activated protein kinase (MAPK) family has been suggested to play a role in apoptosis [33]. Extracellular-signal-regulated kinase (ERK), c-Jun N-terminal kinase (JNK), and p38 MAPK represent the classical type of MAPKs and are activated during various disease conditions. Phosphorylation of MAPK signaling activates MAPKs, which then induce cytokine production [34–37]. The p38 MAPK undergoes dual phosphorylation at Thr182 and Tyr180 in the Thr–Gly–Tyr activation loop by MAP kinase kinase 6 (MKK6) [38–40]. Upon activation, p38 MAPK phosphorylates multiple substrates, including MAPK activated protein kinase 2 (MAPKAPK2) and activating transcription factor 2 (ATF-2) [41, 42]. Heat Shock Protein 27 (HSP27), which is a downstream signaling molecule to MAPKAPK2, is reported to be increased in DENV infection [43]. Upon DENV infection, phosphorylated p38 MAPK increases [20, 44–46]. In addition, DENV induces the phosphorylation of ERK and JNK, and the inhibition of ERK and JNK phosphorylation reduces the infectivity of DENV and protects the liver from injury [45, 47, 48].

SB203580 is a pyridinyl imidazole inhibitor of p38 MAPK, which controls the various inflammatory responses and cellular stresses [26, 49–52]. Interestingly, in human dendritic cells infected with *Zaire ebolavirus*, SB203580 treatment reduces cytokine stimulation [53]. In addition, SB203580 reduces the apoptosis of DENV-infected HepG2 cells by inhibiting RIPK2 expression [17]. The role of p38 MAPK in the apoptosis of DENV-infected HepG2 cells has been investigated, and the DENV-mediated apoptosis of HepG2 cell line is attributed to the increased phosphorylation of p38 MAPK [20]. In a recent study in AG129 immuno-compromised mice (deficient in Type I and II interferon receptors), SB203580 was shown to improve the clinical manifestations of DENV infection. This study shows that oral administration of SB203580 decreased the circulation of pro-inflammatory cytokines. However, the high viral load was still presented in livers of infected mice [54]. In a recent study of intra-cerebral inoculation of DENV in Balb/c mice also shows viremia and the induction of host immune response with organ damage including liver. This study shows the relationship of the elevated pro-inflammatory cytokines in response to DENV infection in Balb/c mice [55]. However, the mechanisms by which SB203580 protects against liver injury during DENV infection *in vivo*, within the complex immune response, need further investigation. Therefore, we further investigated the role of SB203580 in controlling the symptom of DENV-induced liver injury and clarified the molecular mechanism by which SB203580 controls liver injury in a Balb/c mouse model of DENV infection [48].

## Materials and Methods

### DENV-infected mice and SB203580 treatment

Male Balb/c mice, aged 8 weeks and weighing 20–25 g, were purchased from the National Laboratory Animal Center, Mahidol University, Thailand. The animals were strictly maintained under highly sterile conditions, with four mice per cage, at 23 ± 2°C with a 12 h light/dark cycle, and given autoclaved pelleted food and water ad libitum. The animal health was monitored by a veterinarian throughout the study period. All the experiments were conducted in compliance with the institutional policies for animal care and the protocol was approved by the Siriraj Animal Care and Use Committee, Mahidol University (SI-ACUP 004/2556) and the Siriraj Biosafety Risk Management Taskforce, Mahidol University (SI-2013-11). DENV-2 (Thailand strain 16881) propagated in the mosquito cell line C6/36 (purchased from the American Type Culture Collection and further sub-cultured) was used in all experiments. The viral titers were determined with a standard protocol, the Focus Forming Unit (FFU) assay [56]. SB203580 was purchased from Abcam, Cambridge, UK (ab120162) and solubilized in 2% dimethyl sulfoxide (DMSO; Sigma-Aldrich, St. Louis, MO, USA). Mice were randomized into the experiments and were infected intravenously with 4 × 10^5^ FFU/ml of DENV-2 through the lateral tail vein. Twelve mice were infected with DENV, and half of them were treated with the control vehicle, 2%DMSO (n = 6). The other six mice (n = 6) were treated with 5 mg/kg SB203580. Treatments with 2%DMSO alone or SB203580 dissolved in 2%DMSO were given 1 h before and 1 h and 24 h after DENV infection intravenously. In addition, other six mice, which were not infected with DENV, were treated with the control vehicle alone. The volume of all injections was 0.4 ml. At 7 days after infection, the mice were euthanized with an overdose intraperitoneal injection of sodium pentobarbital anesthesia. Intraperitonial injection of sodium pentobarbital anesthesia is applied to minimize animal sufferings. The blood samples for hematological analysis were processed instantly and the sera were prepared for clinical biochemical analysis. Liver tissues were collected and sliced into pieces for analysis. The liver tissues were weighed, and kept frozen in RNALater (Invitrogen, Carlsbad, CA, USA) or RPMI medium for the subsequent isolation of RNA and protein or the FFU assay, respectively. Duplicate independent experiments were performed. Therefore, a total of 36 mice were used in the experiments.

### Quantification of DENV NS1 viral RNA with Real-Time Quantitative Reverse Transcription PCR (qRT-PCR)

*In vitro* transcription-derived DENV NS1 RNA with known copy number is served as a standard control for qRT-PCR [48]. Total RNA was extracted from the livers of 2%-DMSO-treated (n = 6), 2%-DMSO-treated DENV-infected (n = 6), and SB203580-treated DENV-infected mice (n = 6) with the Invitrap Spin Universal RNA Mini Kit (Stratec Molecular) and was quantified with a NanoDrop ND-1000 spectrophotometer. Equivalent amounts of RNA from each sample were converted to cDNA with SuperScript^®^ III First-Strand Synthesis System (Invitrogen) with a reverse primer, NS1-R 5′ GCC ATC AAT GAG AAA GGT CTG G 3′. Amplification was performed using the SYBR Green I reaction mix (Roche) in the presence of NS1 specific primers including NS1-F 5′ CCG GCC AGA TCT GGA GAC ATC AAA GGA ATC 3′ and the NS1-R in a Roche Light Cycler 480. The Ct of viral RNA was measured and compared to the standard control.

### Determination of viral titers in liver homogenates with focus forming unit (FFU) assay

Seven days after DENV infection, the mice were euthanized and the liver tissues were collected from the 2%-DMSO-treated (n = 6), 2%-DMSO-treated DENV-infected (n = 6), and SB203580-treated DENV-infected (n = 6) mice under sterile conditions. The cropped tissues from each groups were equally-weighed. The collected tissues from individual mouse were separately stored in eppendroff tube containing pre-cooled RPMI medium and immediately stored at −80°C. The samples were homogenized in liquid nitrogen with a pestle, and all the samples from each group were centrifuged simultaneously for 5 min at 6,000 × g, and centrifugation was repeated until clear supernatants were obtained. All the centrifugation products were stored at 4°C and the supernatants were filter sterilized. An FFU assay was conducted to determine the viral titers in the centrifuged supernatants, with a previously established protocol [48, 56], and the results were presented as FFU/mg of liver tissue.

### Hematology and measurement of liver enzymes

Blood was collected from the 2%-DMSO-treated (n = 12), 2%-DMSO-treated DENV-infected (n = 12), and SB203580-treated DENV-infected (n = 12) mice, stored in vacutainer tubes containing EDTA, and rapidly processed at the National Laboratory Animal Center, Mahidol University, Thailand. The complete blood count was analyzed with a CELL-DYN^™^ 3700 hematological auto analyzer (Abbott, Abbott Park, IL, USA). To prepare the sera, the blood samples were permitted to clot and then centrifuged. The serum ALT and AST levels were measured with an automated analyzer (Model 902, Hitachi Company, Japan).

### Histopathology

Liver tissues from 2%-DMSO-treated (n = 3), 2%-DMSO-treated DENV-infected (n = 3), and SB203580-treated DENV-infected (n = 3) mice were fixed in 10% formalin in PBS and stored. The fixed tissues were paraffin embedded, sectioned, and conventionally stained with hematoxylin and eosin (H&E).

### Expression of host mRNA with real-time PCR array

The Mouse Apoptosis RT² Profiler^™^ PCR Array (Qiagen, Valencia, CA, USA; cat. # PAMM-012) is a primer preset microarray widely used to analyze the roles of 84 genes involved in apoptosis. RNA was extracted from the liver tissues of the 2%-DMSO-treated, 2%-DMSO-treated DENV-infected, and SB203580-treated DENV-infected mice with the InviTrap Spin Universal RNA Mini Kit (Stratec Molecular). The concentration and purity of the total RNA were measured with a NanoDrop spectrophotometer and same amount of RNA from each group was converted to cDNA with the SuperScript^®^ III First-Strand Synthesis System (Invitrogen). The samples were then mixed with SYBR Green RT^2^ qPCR Mastermix (Qiagen), and equal volumes were aliquoted into each well of the real-time PCR arrays. The Roche LightCycler 480 instrument was used to run the PCR thermal cycling program. The Ct value for each gene was recorded on a Microsoft Excel spreadsheet and the data were uploaded to the web program http://pcrdataanalysis.sabiosciences.com/pcr/arrayanalysis.php for 2^–ΔΔCt^ analysis. The calculated values were presented as fold increases or reductions compared relative to the control group value.

### Cytokine and Chemokine mRNA expression by Real-time RT-PCR

Total RNA from the liver tissues of 2%DMSO treated (N = 3), DMSO treated DENV-infected (N = 3), and DENV-infected SB203580 treated (N = 3) mice was extracted using Invitrap Spin Universal RNA Mini Kit (Stratec Molecular) and converted to cDNA with SuperScript^®^ III First-Strand Synthesis System (Invitrogen). Amplification was continued using specific primers (TNF-α: TNF-F 5’ CCC CCA GTC TGT ATC CTT CT 3’ and TNF-R 5’ TTT GAG TCC TTG ATG GTG GT 3’, IL-6: IL-6-F 5’ AGT TGC CTT CTT GGG ACT GA 3’ and IL-6-R 5’ TCC ACG ATT TCC CAG AGA AC 3’, IL-10: IL-10-F 5’ CCA AGC CTT ATC GGA AAT GA 3’ and IL-10-R 5’ TTT TCA CAG GGG AGA AAT CG 3’, CCL-5: CCL-5-F 5’ CCC TCA CCA TCA TCC TCA CT 3’ and CCL-5-R 5’ CCT TCG AGT GAC AAA CAC GA 3’, CXCL-10: CXCL-10-F 5’ GGA TGG CTG TCC TAG CTC TG 3’ and CXCL-10-R 5’ ATA ACC CCT TGG GAA GAT GG 3’, GAPDH: GAPDH-F 5’ TGA ATA CGG CTA CAG CAA CA 3’ and GAPDH-R 5’ AGG CCC CTC CTG TTA TTA TG 3’) in a Roche Light Cycler 480 using SYBER Green I reaction mix (Roche). Threshold Ct value of each gene of interest and GAPDH (housekeeping gene) were measured and the differences between the gene of interest to the GAPDH (ΔCt) were calculated. The relative expression values (2^-ΔΔCt^) were then determined. Results were obtained from three independent experiments for three independent mice per individual group.

### Western blotting analysis

Total protein was extracted from the liver tissues of the 2%-DMSO-treated, 2%-DMSO treated DENV-infected, and SB203580-treated DENV-infected mice in RIPA buffer containing a protease inhibitor (Roche), and was subjected to western blotting analysis [57]. To detect the phosphorylated proteins, a phosphatase inhibitor cocktail (Roche) was also added. The concentrations of the extracted proteins were estimated with a Bradford Protein Assay (Bio-Rad Laboratories, Inc. Hercules, CA, USA). Equivalent protein concentrations from the individual groups were mixed with 4× loading dye (50 mM Tris-HCl [pH 6.8], 2% SDS, 0.1% bromophenol blue, and 10% glycerol) and denatured at 95°C for 5 min in a thermocycler. The samples were separated with SDS-PAGE and blotted onto nitrocellulose membrane (GE Healthcare Life Sciences, Freiburg, Germany). The membrane was blocked with 5% bovine serum albumin or 5% skim milk in Tris-buffered saline containing 0.1% Tween 20 (TBST) to restrict nonspecific binding. The membrane was washed three times and hybridized with rabbit anti-total p38 MAPK (Santa Cruz Biotechnology, Santa Cruz, CA, USA) at a dilution of 1:2000, or mouse anti-phosphorylated p38 MAPK (Santa Cruz Biotechnology) at a dilution of 1:1000, or rabbit anti-total HSP27 (Cell Signaling Technology, MA, USA) at a dilution of 1:1000, or rabbit anti-phosphorylated HSP27 (Cell Signaling) at a dilution of 1:1000, or rabbit anti-total MAPKAPK2 (Santa Cruz Biotechnology) at a dilution of 1:1000, or rabbit anti-phosphorylated MAPKAPK2 (Cell Signaling Technology) at a dilution of 1:2000, or rabbit anti-total ATF2 (Santa Cruz Biotechnology) at a dilution of 1:1000, or mouse anti-phosphorylated ATF2 (Santa Cruz Biotechnology) at a dilution of 1:1000, or goat anti-TNF-α (Santa Cruz Biotechnology) at a dilution of 1:2000, or rabbit anti-caspase 8 (Santa Cruz Biotechnology) at a dilution of 1:1000, or mouse anti-caspase 9 (Cell Signaling Technology) at a dilution of 1:1000, or rabbit anti-GGT1 antibody (Santa Cruz Biotechnology) at a dilution of 1:2500, or rabbit anti-GAPDH (Cell Signaling Technology) at a dilution of 1:2500, or rabbit anti-cleaved caspase 3 antibody (Cell Signaling Technology) at a dilution of 1:2000. The membrane was washed three times with TBST and incubated for 1 h in the dark at room temperature with horseradish peroxidase (HRP)-conjugated secondary antibody, HRP-conjugated rabbit anti-mouse IgG antibody for phosphorylated p38 MAPK, phosphorylated ATF2 and Caspase 9 (Dako, Santa Clara, CA, USA), HRP-conjugated rabbit anti-goat IgG antibody for TNF-α (Dako, Santa Clara, CA, USA), or HRP-conjugated swine anti-rabbit IgG antibody for the all other proteins (Dako, Santa Clara, CA, USA). The membranes were washed three times with TBST in the dark and the immune complexes were detected with enhanced chemiluminescence (SuperSignal West Pico Chemiluminescent Substrate; Thermo Scientific, Waltham, MA, USA). GAPDH is used as the housekeeping gene for all the western blot analysis.

### Statistical analysis

For the estimation of hematology and liver enzymes, the results were pooled from the two independent experiments (total of twelve mice per group from two independent experiments). For the quantification of viral NS1, a total of six mice per group were used. For the all other experiments, at least three independent experiments from three independent mice per individual group (n = 3) were performed. All the results were presented as means ± SEM and the data were analyzed using the GraphPad Prism Software version 5. Statistical analysis was conducted by One-way ANOVA and p value less than 0.05 were considered as statistically significant (*p* < 0.05).

## Results

### SB203580 treatment modulates hematological parameters in DENV-infected mice

**Fig 1:**
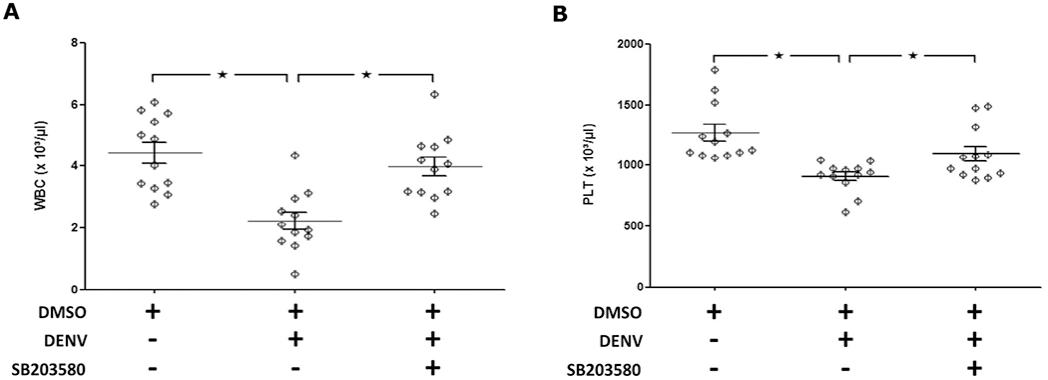
The hematological phenotype of the DENV-infected mice was improved by SB203580 treatment. Mice were infected with DENV and treated with 2%DMSO or SB203580. The uninfected control group was also treated with 2%DMSO alone. Seven days after infection, their blood was collected in EDTA tubes for hematological analysis. (A) WBC counts, and (B) platelet counts. The results were pooled from two independent experiments, and are presented as means ± SEM (a total of 12 mice per group from two independent experiments). The asterisks indicate statistically significant differences between groups (*p* < 0.05).

In this study, Balb/c mice were intravenously injected with the vehicle control alone (2%DMSO) or were infected with DENV and treated with 2%DMSO or SB203580. All mice survived the challenge protocol, with no any clinical symptoms observed till the end of the protocol. However, the white blood cell (WBC) and platelet counts decreased significantly in the DENV-infected mice, suggesting leucopenia and thrombocytopenia, respectively. The hematological phenotype of the Balb/c mice was improved significantly by SB203580 treatment (Fig 1A and 1B).

### SB203580 treatment reduces liver injury in DENV-infected mice

**Fig 2:**
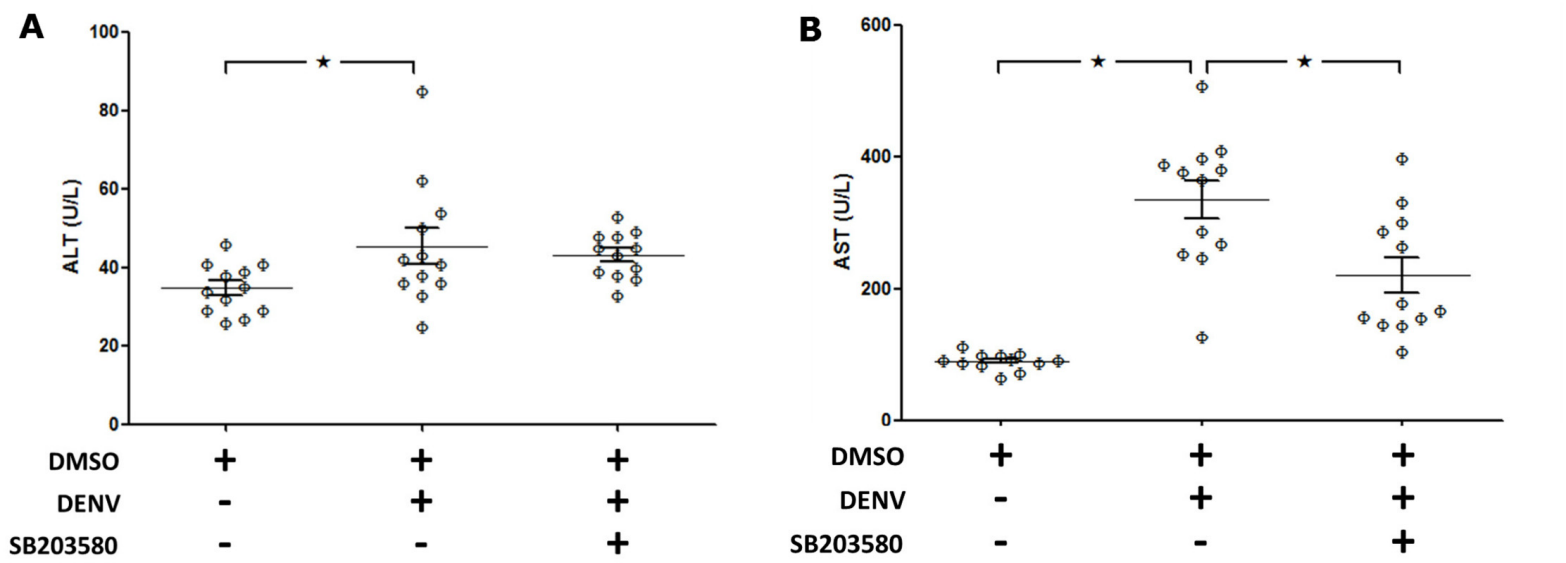
SB203580 treatment reduces liver enzymes in DENV-infected mice. Mice were infected with DENV and treated with 2%DMSO or SB203580. The uninfected control group was also treated with 2%DMSO. Seven days after infection, blood was collected and sera prepared to estimate liver transaminases. (A) ALT, and (B) AST. The pooled results of two independent experiments are presented as means ± SEM (12 mice per group in two independent experiments). The asterisks indicate statistically significant differences between the groups (*p* < 0.05).

Mice were intravenously infected with DENV, and their liver transaminases ALT and AST were found to be significantly increased compared with those of the uninfected control group (Fig 2A and 2B) on day 7. However, after the DENV-infected mice were treated with SB203580, their liver AST levels were significantly reduced, but the changes in ALT were failed to reach statistical significance (Fig 2A and 2B).

**Fig 3:**
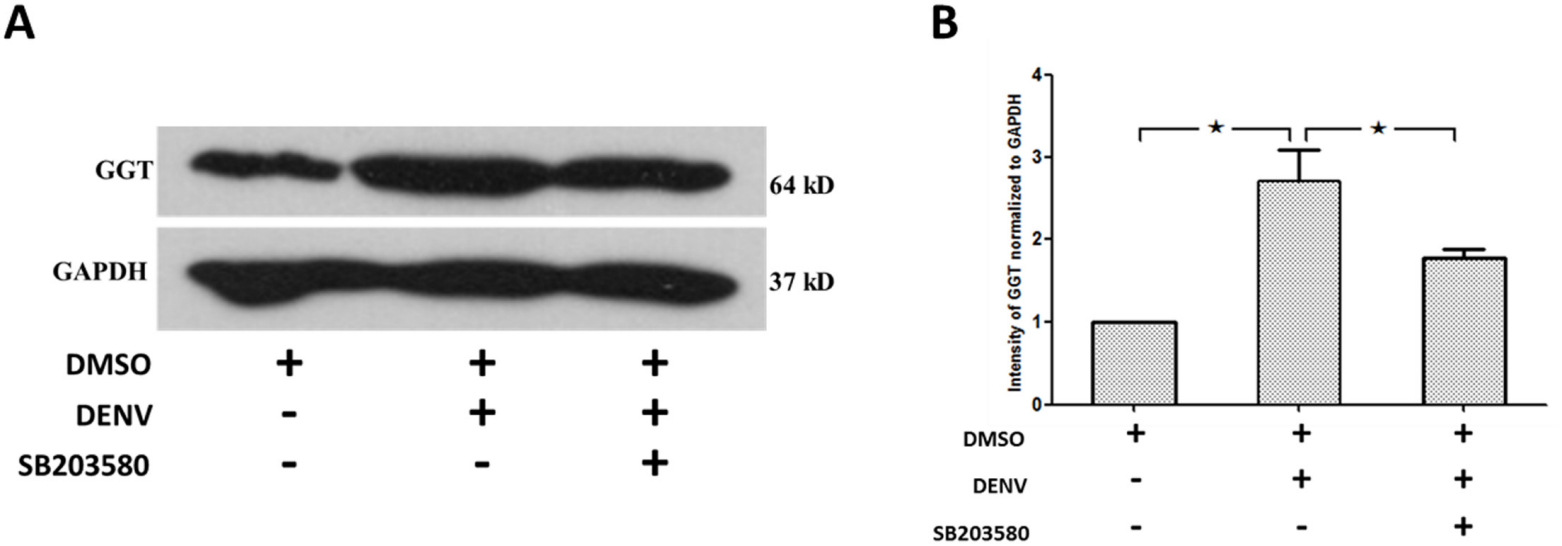
SB203580 treatment reduces GGT expression in DENV-infected mice. Mice were infected with 4 × 10^5^ FFU/ml of DENV and treated with 2%DMSO (v/v) or SB203580 dissolved in 2%DMSO. The control (uninfected) group was treated with 2%DMSO (v/v) alone. Treatments were given 1 h before and after DENV infection and again at 24 h after infection. On day 7 after infection, the liver tissues were collected, and the proteins were extracted for western blotting analysis using antibody directed against (A) GGT normalized to GAPDH. The results shown are representatives of three independent experiments with three mice (n = 3) from each group. A densitometry analysis using the ImageJ software is shown in (B).

Gamma-glutamyl transpeptidase (GGT) is reported as a biomarker for hepatocellular and cholestatic damage [58]. Elevated level of GGT was observed in various liver injuries [59–62]. Therefore, western blot analysis with antibody directed against GGT was conducted and normalized to GAPDH. The results show that the expression of GGT in liver of 2%DMSO treated DENV-infected mice was significantly higher than that of 2%DMSO treated mice (Fig 3A). Intravenous treatment of SB203580 reduced the GGT expression in livers of DENV-infected mice suggesting the decreased liver injury in SB203580 treated mice. Three independent experiments were conducted and densitometry analysis was conducted using ImageJ for three independent mice from each group (Fig 3B).

**Fig 4:**
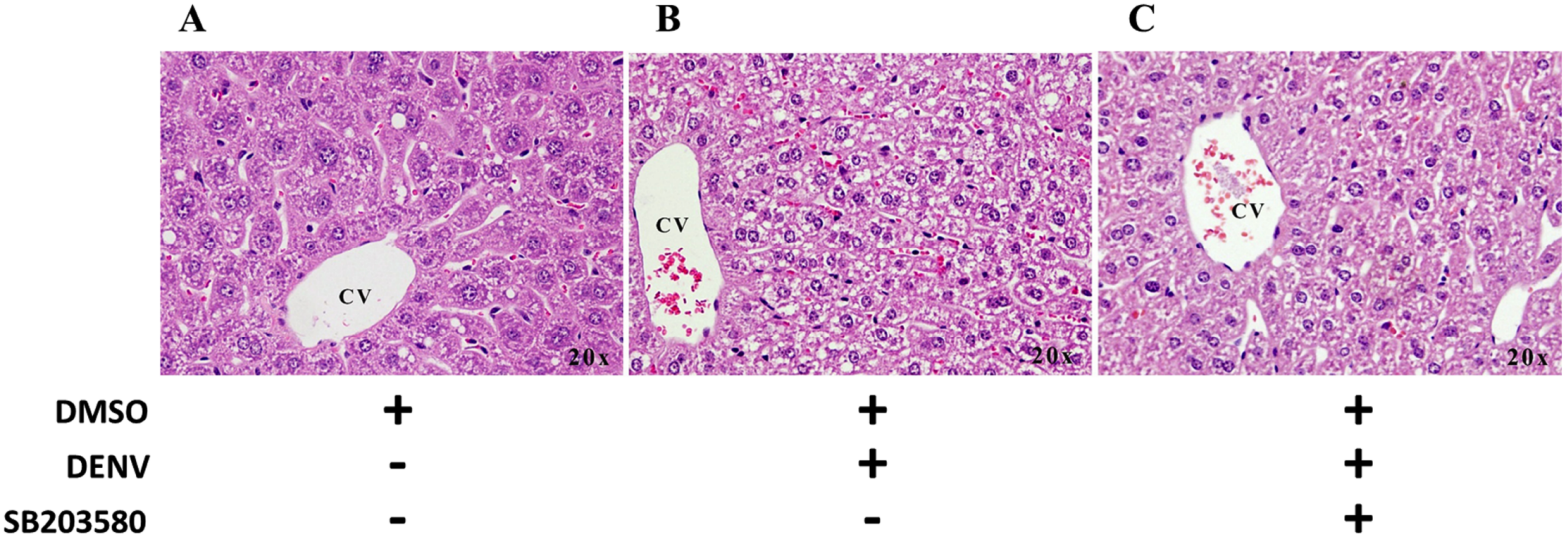
SB203580 treatment reduces DENV-induced liver pathology in DENV-infected mice. Mice were infected with DENV and treated with 2%DMSO or SB203580 dissolved in 2%DMSO. The uninfected control group was also treated with 2%DMSO. Seven days after infection, liver tissues were collected in 10% formalin for histopathological analysis and H&E staining. (A) 2%DMSO alone (B) 2%DMSO-treated DENV-infected (C) SB203580-treated DENV-infected. The results shown are representative of ≥ 3 mice from each group.

Histological analysis of the liver tissues of the uninfected 2%DMSO-treated mice (Fig 4A), 2%DMSO-treated DENV-infected mice (Fig 4B), and SB203580-treated DENV-infected mice (Fig 4C) was conducted. The 2%DMSO-treated DENV-infected mice showed classical signs of liver injury, with dilated sinusoid capillaries and numerous hyperplastic Kupffer cells around them, and the hepatocytes were enlarged by cytoplasmic vacuolization. Specifically, marked cellular necrosis and apoptosis were also noted in a histological analysis of the 2%DMSO-treated DENV-infected mice. Interestingly, the SB203580-treated DENV-infected mice showed less liver injury than that of the 2%DMSO-treated DENV-infected mice.

### SB203580 does not reduce dengue virus production

**Fig 5:**
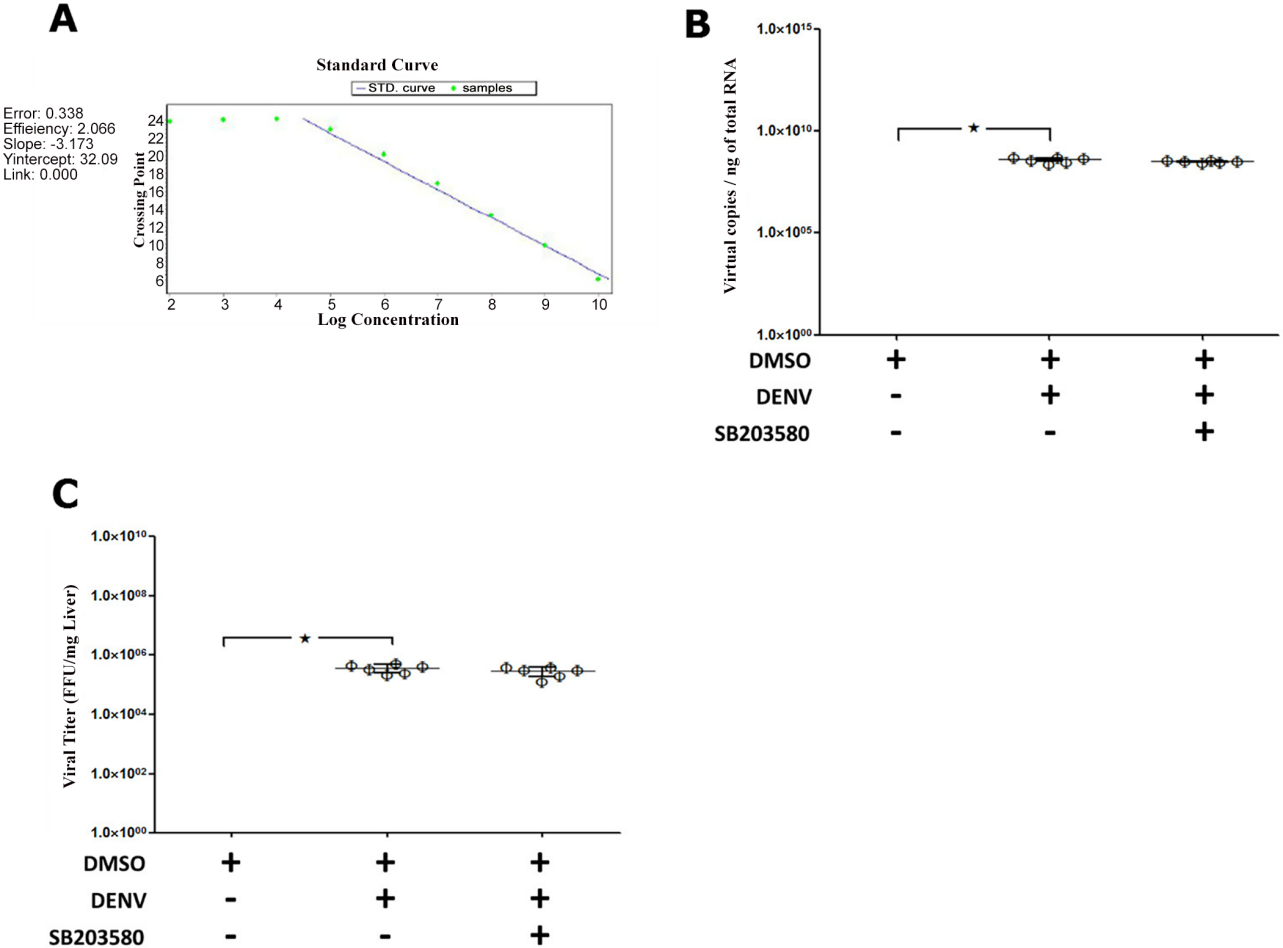
SB203580 does not reduce DENV production in DENV-infected mice. Mice were infected with 4 × 10^5^ FFU/ml of DENV and treated with 2%DMSO alone or SB203580 in 2%DMSO. The uninfected control group was also treated with 2%DMSO. Seven days after infection, the liver tissues were collected, preserved in RNALater for the quantification of viral NS1, and in RPMI medium for an FFU assay. Total RNA was extracted to quantify viral NS1 with qRT–PCR. (A) Standard curve plotted from the Ct values for 10-fold serially diluted cDNA of known copy number (10^1^–10^10^ copies). The dots represent the Ct values of each 10-fold dilution. (B) Viral NS1 copies in 1 μg of total RNA from 2%DMSO-treated (uninfected), 2%DMSO-treated DENV-infected, and SB203580-treated DENV-infected groups of mice (viral copies/μg). The supernatant was prepared from the liver tissue homogenates for the FFU assay. (C) Viral titers in the 2%DMSO-treated (uninfected), 2%DMSO-treated DENV-infected, and SB203580-treated DENV-infected groups of mice are expressed in FFU per milligram (FFU/mg). The results were obtained from six animals per group (n = 6). The data were analyzed with One-way ANOVA using GraphPad Prism 5 and are presented as means ± SEM. Asterisks show the level of significance (*p* < 0.05 is considered statistically significant).

To test whether SB203580 treatment modulates dengue virus production or host immune responses, the viral RNA copies in the liver of mice from each group were quantified. A known copy number of *in vitro*-transcribed DENV NS1 was used as the standard control PCR template. A standard curve was plotted with the Ct values for known copy numbers of DENV NS1, as shown in Fig 5A. The RNA from the liver tissue homogenates of mice treated with 2%DMSO alone, 2%DMSO-treated DENV-infected mice, and SB203580-treated DENV-infected mice was prepared and subjected to qRT–PCR with primers specific for DENV NS1. When six mice from each group were analyzed, 1 μg of total RNA from the 2%DMSO-treated DENV-infected mice contained an average of 3.843 × 10^8^ DENV NS1 copies and that from SB203580-treated DENV-infected mice contained 3.147 × 10^8^ DENV NS1 copies (Fig 5B). No DENV NS1 copies were detected in any mouse not infected with DENV and treated with 2%DMSO alone. The viral FFUs in the liver homogenates were determined with the FFU assay. The liver tissues from the 2%DMSO-alone-treated, 2%DMSO-treated DENV-infected, and SB203580-treated DENV-infected mice were collected on day 7 in RPMI medium. The homogenates were prepared and centrifuged to obtain the supernatants, which were analyzed with FFU assay. There was no significant difference of in the liver homogenate FFUs of the SB203580-treated DENV-infected mice and those of 2%DMSO treated DENV-infected mice (Fig 5C).

### SB203580 treatment modulates the apoptotic gene expression profile in DENV-infected mice

To explore the molecular mechanism by which SB203580 reduces liver damage, screening experiments were conducted with a commercially available Mouse Apoptosis RT^2^ Profiler^™^ PCR Array System (Qiagen). Total RNA was isolated from the 2%DMSO-alone group, 2%DMSO-treated DENV-infected group, and SB203580-treated DENV-infected group, converted to cDNA, and amplified with the LightCycler 480 PCR system. The full list of gene expression profile of the apoptosis related genes in DENV-infected mice and the effect of SB203580 treatment to those genes were separately shown in S1 Table. Table 1 lists the gene expression profile of the selected apoptosis related genes whose expression is increased in 2%DMSO-treated DENV infected mouse and reduced by SB203580-treated DENV-infected mouse, which is normalized to un-infected 2%DMSO treated mouse. Actin is used as the housekeeping gene for normalizing the expression profile.

**Table 1:** Apoptotic gene expression profiles of DMSO-treated DENV-infected mice and SB203580 treated DENV-infected mice (both are normalized with those of DMSO-treated uninfected mice).

Gene	Gene Description	DMSO+DENV	DENV+SB203580
Apaf1	Apoptotic peptidase activating factor 1	2.5669	1.8404
Bax	Bcl2-associated X protein	3.3173	1.1729
Bcl2a1a	B-cell leukemia/lymphoma 2 related protein A1a	11.3924	6.4082
Casp1	Caspase 1	4.1411	5.4642
Casp12	Caspase 12	4.7899	2.1737
Casp14	Caspase 14	2.5491	1.1407
Casp2	Caspase 2	2.4284	1.8531
Casp3	Caspase 3	2.1886	1.8277
Casp8	Caspase 8	2.1140	1.9588
Casp9	Caspase 9	1.9588	1.1647
Cd40	CD40 antigen	3.0314	1.3850
Cd40lg	CD40 ligand	1.7411	0.5323
Cidea	Cell death-inducing DNA fragmentation factor, alpha subunit-like effector A	8.3977	2.3295
Cradd	CASP2 and RIPK1 domain containing adaptor with death domain	4.6268	2.4286
Fadd	Fas (TNFRSF6)-associated via death domain	2.4623	2.0705
Fasl	Fas ligand (TNF superfamily, member 6)	11.9588	7.8904
Il10	Interleukin 10	10.1261	1.0278
Pycard	PYD and CARD domain containing	3.3870	1.7413
Tnf	Tumor necrosis factor	2.5491	1.1407
Tnfsf10	Tumor necrosis factor (ligand) superfamily, member 10	11.3924	3.8370

### SB203580 treatment reduces apoptosis

**Fig 6:**
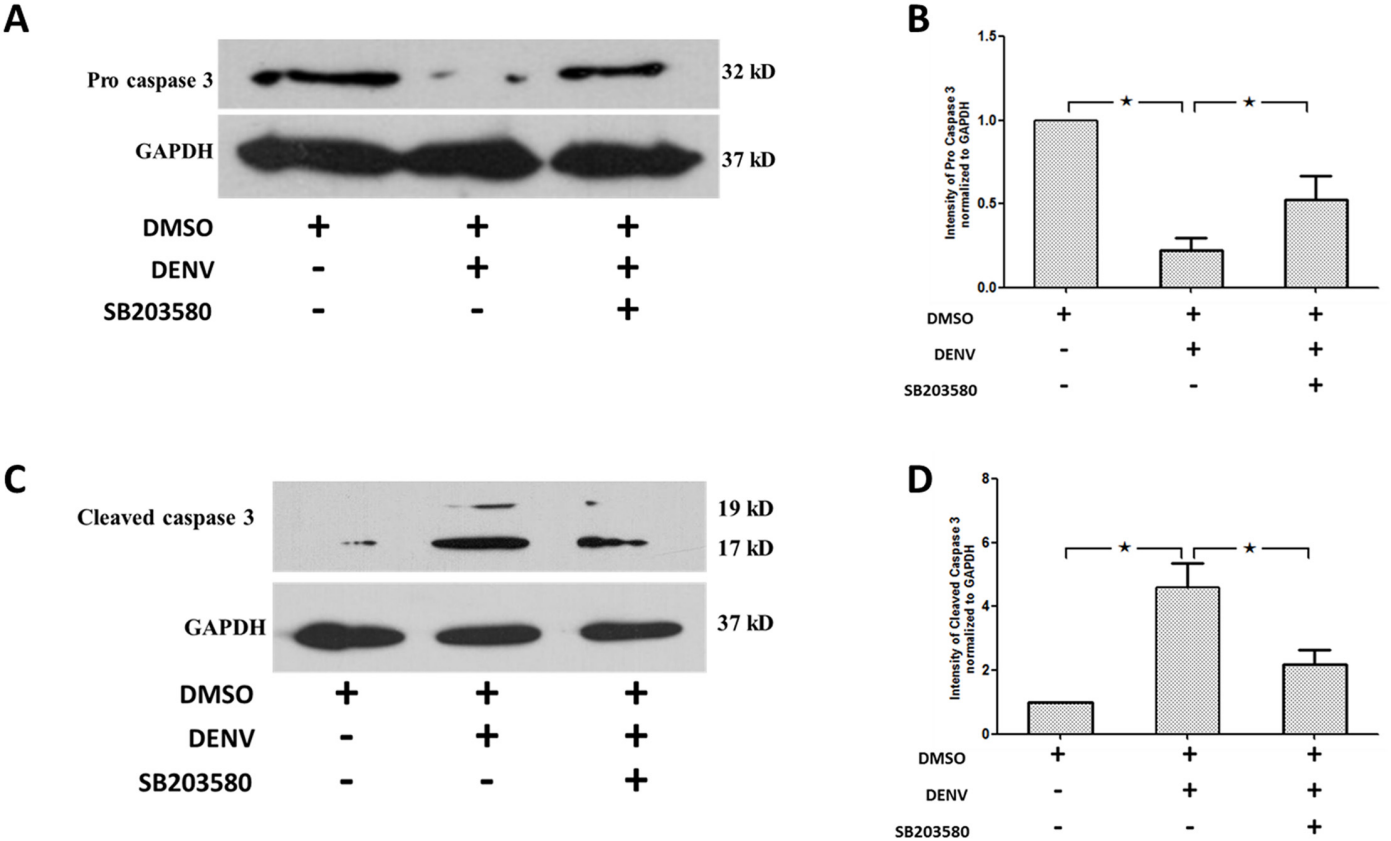
SB203580 treatment reduces caspase 3 expression. Mice were infected with 4 × 10^5^ FFU/ml of DENV and treated with 2%DMSO (v/v) or SB203580 dissolved in 2%DMSO. The control (un-infected) group was treated with 2%DMSO (v/v) alone. Treatments were given 1 h before and after DENV infection and again at 24 h after infection. On day 7 after infection, the liver tissues were collected, and the proteins were extracted for western blotting analysis using antibodies directed against (A) pro caspase 3 and (C) cleaved caspase 3. The results shown are representative of three independent experiments with three mice (n = 3) from each group. A densitometry analysis using the ImageJ software is shown in (B) pro caspase 3 and (D) cleaved caspase 3.

We confirmed the efficacy of SB203580 in modulating apoptosis in DENV-infected mice by western blot analysis. The expression of both pro and cleaved forms of caspase 3 in un-infected 2%DMSO treated, 2%DMSO-treated DENV-infected and SB203580 treated DENV-infected mice were evaluated. The pro-caspase 3 was decreased in 2%DMSO-treated DENV-infected mice (Fig 6A and 6B) compared to that of un-infected 2%DMSO-treated mice. Interestingly, SB203580-treated DENV-infected mice show significant increase in the pro-caspase 3. To further verify the results, an antibody that detects the cleaved form of caspase 3 was used in western blot analysis. The results show an increased expression of cleaved caspase 3 expression in the 2%DMSO-treated DENV-infected mice (Fig 6C and 6D) compared to that of un-infected 2%DMSO-treated control mice. A significant reduction in the expression of cleaved caspase 3 was observed in SB203580 treated DENV-infected mice (Fig 6C and 6D). Our results confirm that SB203580 treatment in DENV-infected mice restrict the cleavage of caspase 3 thereby reducing cellular apoptosis.

**Fig 7:**
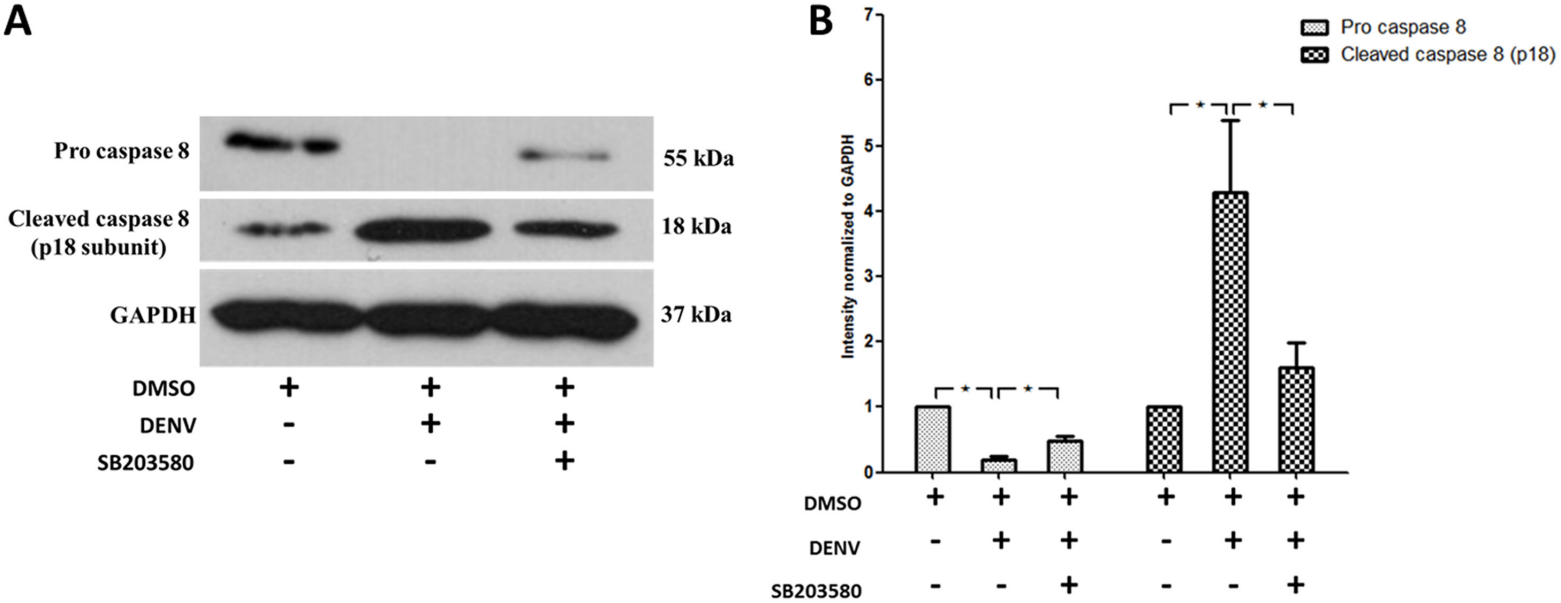
SB203580 treatment reduces caspase 8 expression. Mice were infected with 4 × 10^5^ FFU/ml of DENV and treated with 2%DMSO (v/v) or SB203580 dissolved in 2%DMSO. The control (uninfected) group was treated with 2%DMSO (v/v) alone. Treatments were given 1 h before and after DENV infection and again at 24 h after infection. On day 7 after infection, the liver tissues were collected, and the proteins were extracted for western blotting analysis using antibody directed against the pro and p18 subunit of the cleaved form of (A) caspase 8. The results shown are representative of three independent experiments with three mice (n = 3) from each group. A densitometry analysis using the ImageJ software is shown in (B).

To further characterize in what way the apoptosis is controlled by SB203580 in DENV infected mice, we explored the specific markers for extrinsic and intrinsic apoptosis pathways. To characterize the extrinsic pathway, an antibody that detects both pro caspase 8 and cleaved caspase 8 was used for western blot analysis. Pro-caspase 8 in the 2%DMSO-treated DENV-infected mice was significantly less to that of un-infected mice treated with 2%DMSO alone (Fig 7A), whereas SB203580 treatment reversed the pro-caspase 8 of DENV-infected mice (Fig 7A). As expected, cleaved caspase 8 was activated in the 2%DMSO-treated DENV-infected mice (Fig 7A), whereas treatment with SB203580 reduced the cleaved caspase 8 expression (Fig 7A). Our results confirm that caspase 8 is activated in DENV infection and SB203580 treatment significantly restricts this activation, suggesting the role of SB203580 in reduction of the extrinsic pathway of apoptosis.

**Fig 8:**
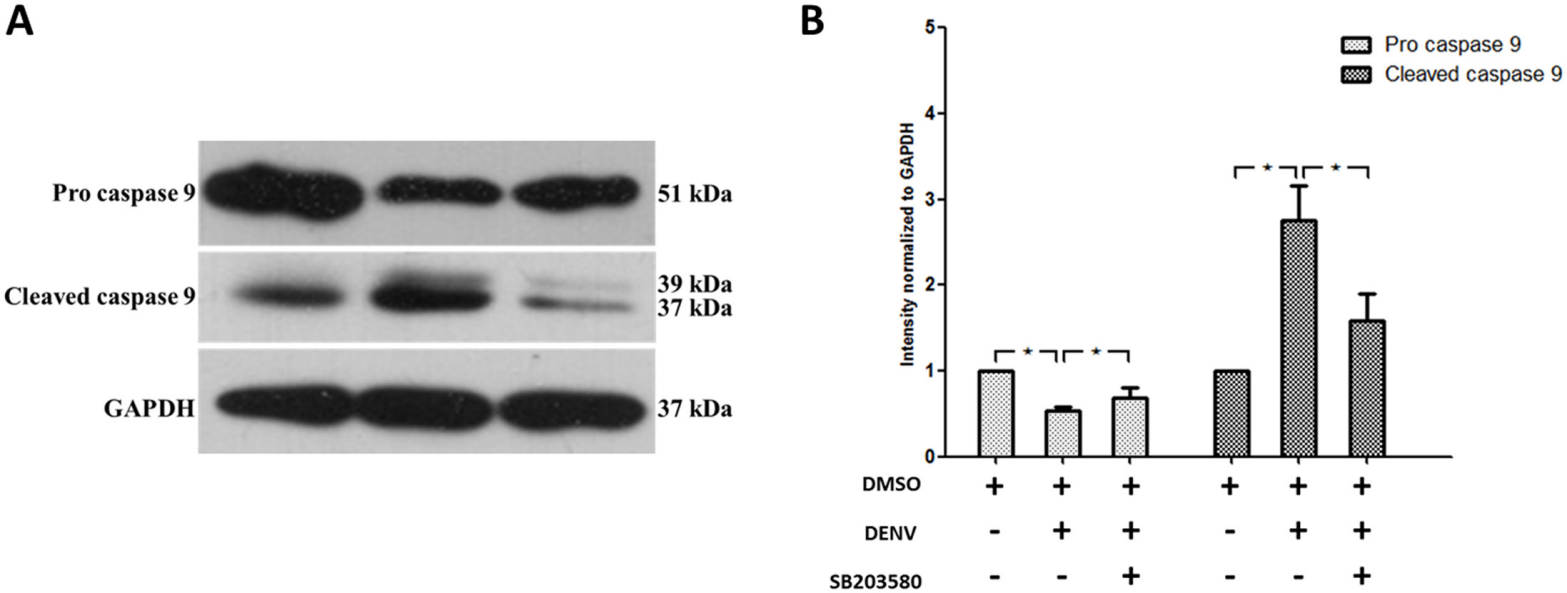
SB203580 treatment reduces caspase 9 expression. Mice were infected with 4 × 10^5^ FFU/ml of DENV and treated with 2%DMSO (v/v) or SB203580 dissolved in 2%DMSO. The control (uninfected) group was treated with 2%DMSO (v/v) alone. Treatments were given 1 h before and after DENV infection and again at 24 h after infection. On day 7 after infection, the liver tissues were collected, and the proteins were extracted for western blotting analysis using antibodies directed against the pro and cleaved forms of (A) caspase 9. The results shown are representative of three independent experiments with three mice (n = 3) from each group. A densitometry analysis using the ImageJ software is shown in (B).

We further investigated the role of intrinsic pathway using an antibody against caspase 9, one of the classical markers for mitochondrial cell death. Pro caspase 9 was significantly reduced in 2%DMSO treated DENV infected mice compared to that of un-infected 2%DMSO control mice. SB203580 reversed the pro caspase 9 expression in DENV infected mice (Fig 8A). Interestingly, the cleaved caspase 9 was significantly higher in the 2%DMSO-treated DENV-infected mice than that of un-infected 2%DMSO treated control mice and SB203580 treatment reduced the cleaved caspase 9 expression in DENV infected mice (Fig 8A). The results shown are representative of three independent experiments with three mice (n = 3) from each group. A densitometry analysis using the ImageJ software is shown in (Fig 8B). Our results show that the reduced pro caspase 9 in 2%DMSO-treated DENV infected mice was found, as it is cleaved to 37 and 39 sub units of cleaved caspase 9 and SB203580 treatment restricted this cleavage and modulated the caspase 9 expression in DENV-infected mice. Specifically, our results confirm that SB203580 modulate the intrinsic pathway of apoptosis in DENV-infected mice.

### SB203580 treatment modulates the cytokine and chemokine gene expression profile in DENV-infected mice

**Fig 9:**
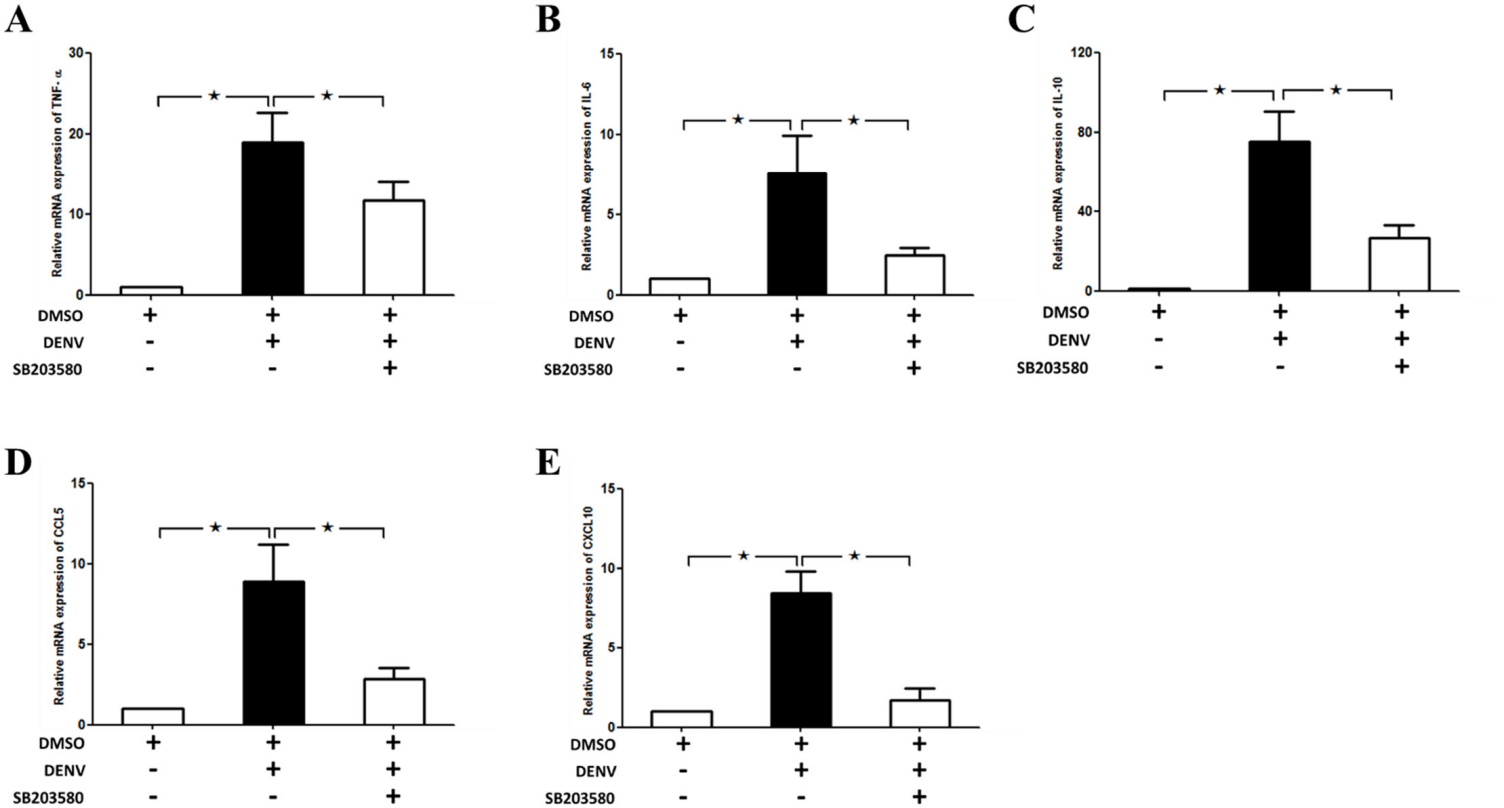
SB203580 modulates the cytokine and chemokine gene expressions in DENV infection Mice were infected with 4 × 10^5^ FFU/ml of DENV and treated with 2%DMSO (v/v) or SB203580 dissolved in 2%DMSO. The control (uninfected) group was treated with 2%DMSO (v/v) alone. Treatments were given 1 h before and after DENV infection and again at 24 h after infection. On day 7 after infection, the liver tissues were collected, and RNA was extracted, cDNA were prepared and which undergone Real-time RT PCR analysis with individual primer set. GAPDH is used as the house keeping gene. The mRNA expression of (A) TNF-α (B) IL-6 (C) IL-10 (D) CCL-5 (E) CXCL-10 are shown. Results were represented in the graph by three independent experiments for at least 3 independent mice from each group. Statistical analysis is conducted by One Way ANOVA using GraphPad Prism Software Version 5. The asterisks indicate statistically significant differences between groups (*p*< 0.05).

The mRNA expression of cytokines including Tumor Necrosis Factor-α (TNF-α), IL-6 and IL-10, chemokines including CCL-5 (RANTES) and CXCL-10 (IP-10) were analyzed by RT-PCR. An mRNA expression of GAPDH is used to normalize the cytokine and chemokine expressions. The results were expressed in fold times to that of un-infected 2%DMSO control. DENV-infection resulted with significant up-regulation of cytokines and chemokines in DENV-infected mice on Day 7. Our results show up-regulated expression of cytokines including TNF-α (Fig 9A), IL-6 (Fig 9B) and IL-10 (Fig 9C) in 2%DMSO-treated DENV-infected mice and SB203580 treatment significantly reduced the TNF-α, IL-6 and IL-10 expression upon DENV-infection (Fig 9A, 9B and 9C). Similarly, the chemokines including CCL-5 (Fig 6D) and CXCL-10 (Fig 9E) were also up-regulated in 2%DMSO-treated DENV-infected mice and SB203580 treatment significantly reduced the mRNA expression of RANTES and IP-10 (Fig 9D and 9E). Interestingly, our results suggest the efficacy of SB203580 in controlling the cytokine and chemokine expression in DENV-infected mice.

**Fig 10:**
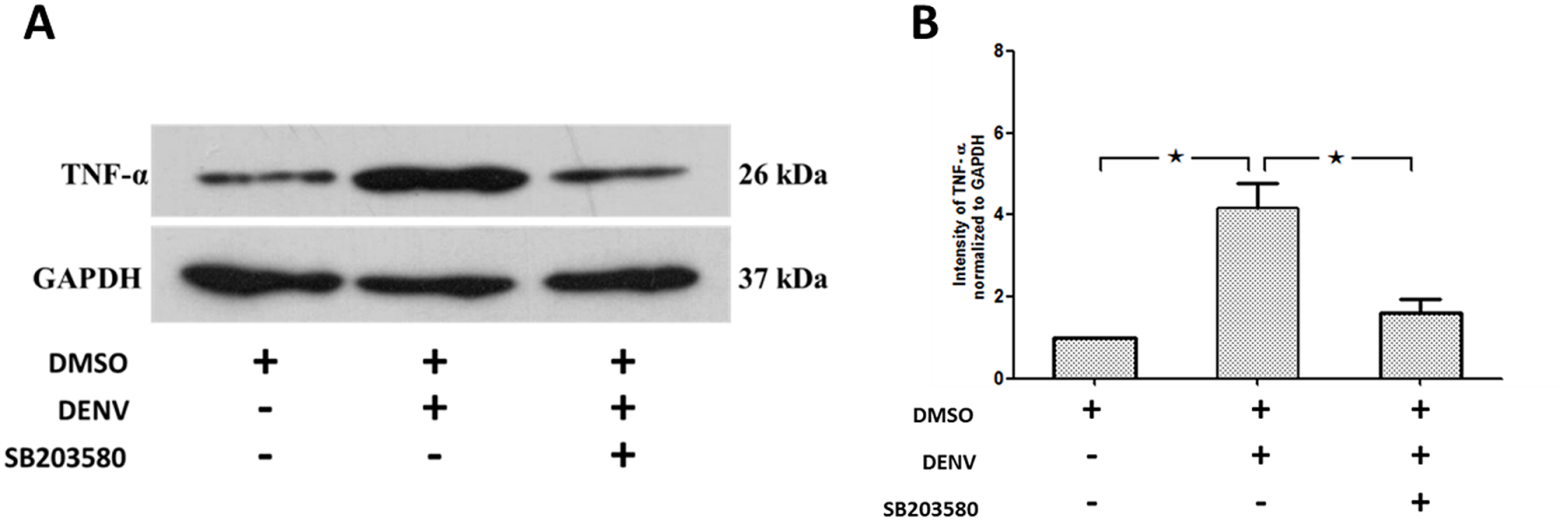
SB203580 treatment reduces TNF-α in DENV-infected mice. Mice were infected with 4 × 10^5^ FFU/ml of DENV and treated with 2%DMSO (v/v) or SB203580 dissolved in 2%DMSO. The control (uninfected) group was treated with 2%DMSO (v/v) alone. Treatments were given 1 h before and after DENV infection and again at 24 h after infection. On day 7 after infection, the liver tissues were collected, and the proteins were extracted for western blotting analysis using antibodies directed against (A) TNF-α. The results shown are representative of three independent experiments with three mice (n = 3) from each group. A densitometry analysis using the ImageJ software is shown in (B).

To validate the RT-PCR analysis, we investigated the protein expression of the prominent pro-inflammatory cytokine, TNF-α, in the liver tissue by western blot analysis. As expected, increased expression of TNF-α was observed in the 2%DMSO-treated DENV-infected mice (Fig 10A) compared to that of un-infected 2%DMSO treated mice and SB203580 significantly reduced the TNF-α production in the DENV-infected mice (Fig 10A). The results shown are representative of three independent experiments with three mice (n = 3) from each group. A densitometry analysis using the ImageJ software is shown in Fig 10B.

### SB203580 does not reduce the phosphorylation of p38 MAPK

**Fig 11:**
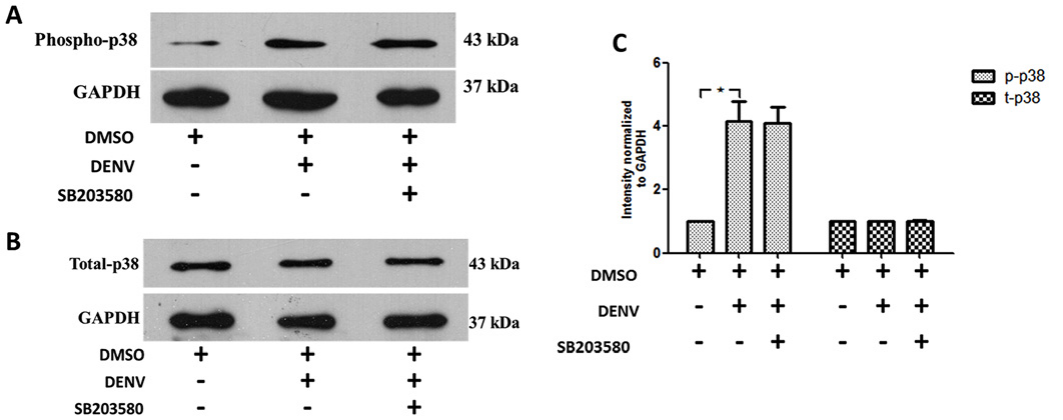
SB203580 does not reduce the phosphorylation of p38 MAPK. Proteins were extracted from the liver tissue samples of 2%DMSO-treated (uninfected), 2%DMSO-treated DENV-infected, and SB203580-treated DENV-infected groups of mice. An additional cocktail of phosphatase inhibitors was added for maintaining the phosphorylated proteins and subjected to western blot analysis with specific antibodies. Results were shown for (A) phosphorylated p38 MAPK and (B) total p38 MAPK. The results shown are representative of three independent experiments with three mice (n = 3) from each group. A densitometry analysis was conducted for the individual blots, normalized to the respective GAPDH, is shown in (C) phosphorylated p38 MAPK and total p38MAPK.

To explore the molecular mechanism by which the p38 MAPK inhibitor, SB203580 involves in the apoptotic pathways, we firstly tested whether DENV induces the phosphorylation of p38 MAPK in DENV-infected mice and further the ability of SB203580 to reduce the phosphorylated p38 MAPK. Our results show that DENV infection induced the phosphorylation of p38 MAPK and the treatment of with SB203580 in DENV-infected mice caused no reduction of p38 MAPK phosphorylation *in vivo* (Fig 11A). The total p38 MAPK remains equal in all group of mice (Fig 11B). The results were normalized to the house keeping gene GAPDH. The results were representative of at least three independent experiment obtained from three animals per group (n = 3). Densitometry analysis was conducted using ImageJ software and shown in Fig 11C.

### SB203580 reduces phosphorylation of the downstream signaling molecules to p38 MAPK

**Fig 12:**
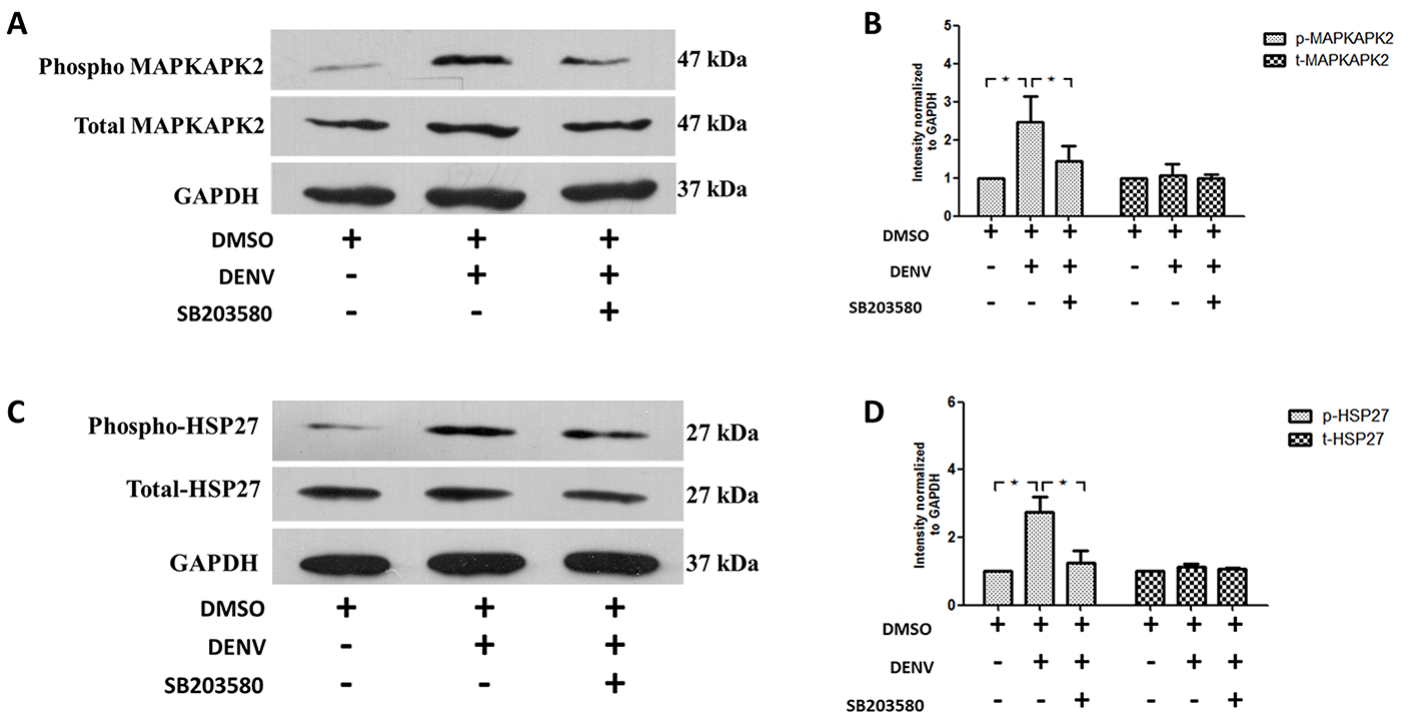
SB203580 treatment reduces the phosphorylation of MAPKAPK2 and HSP27. Proteins were extracted from the liver tissue samples of 2%DMSO-treated (un-infected), 2%DMSO-treated DENV-infected, and SB203580-treated DENV-infected groups of mice. An additional cocktail of phosphatase inhibitors was added for maintaining the phosphorylated proteins and allowed them to standard Western blot analysis with specific antibodies. The results were shown (A) phosphorylated MAPKAPK2 and total MAPKAPK2 and (C) phosphorylated HSP27 and total HSP27, which is normalized to their respective GAPDH. The results shown are representative of three independent experiments with three mice (n = 3) from each group. A densitometry analysis was conducted with the individual blots normalized to GAPDH and is shown in (B) phosphorylated MAPKAPK2 and total MAPKAPK2 and (D) phosphorylated HSP27 and total HSP27.

Our results show that SB203580 treatment does not reduce the phosphorylated p38 MAPK in DENV-infected mice, we further examined how SB203580 reduces apoptosis in DENV-infected mice. We hypothesized whether the phosphorylation of any of the downstream signaling molecules to p38 MAPK is controlled by SB203580 treatment. We investigated whether DENV infection induces the phosphorylation of MAPKAPK2, the downstream target to p38 MAPK. Western blot analysis with antibodies directed against phosphorylated MAPKAPK2 and total MAPKAPK2 was conducted. We found that DENV infection induces phosphorylation of MAPKAPK2 (Fig 12A) and SB203580 is able to restrict the MAPKAPK2 phosphorylation. Our results confirm SB203580 treatment reduces the phosphorylated form of MAPKAPK2 in DENV infected mice (Fig 12A). The total MAPKAPK2 remains equal in each group of mice (Fig 12A). We suggest that the reduced phosphorylation of MAPKAPK2 by SB203580 treatment contributes to the reduced apoptosis in DENV-infected mice. We further investigated the downstream signaling molecules to MAPKAPK2, namely HSP-27. Western blot analysis with antibodies directed against phosphorylated HSP27 and total HSP27 was conducted. Our results show that DENV infection induces the phosphorylation of HSP27 (Fig 12C) in DENV-infected mice and SB203580 treatment reduces the phosphorylation of HSP27 (Fig 12C). The total HSP-27 remains equal in each group of mice (Fig 12C). Interestingly, we suggest that SB203580 didn’t reduce the phosphorylation of p38 MAPK, but blocks the phosphorylation of its downstream signals including MAPKAPK2 and HSP-27. Individual blots were normalized to individual GAPDH. The results were representative of at least three independent experiment obtained from three animals per group (n = 3). Densitometry analysis was conducted using ImageJ software and represented in Fig 12B and 12D.

**Fig 13:**
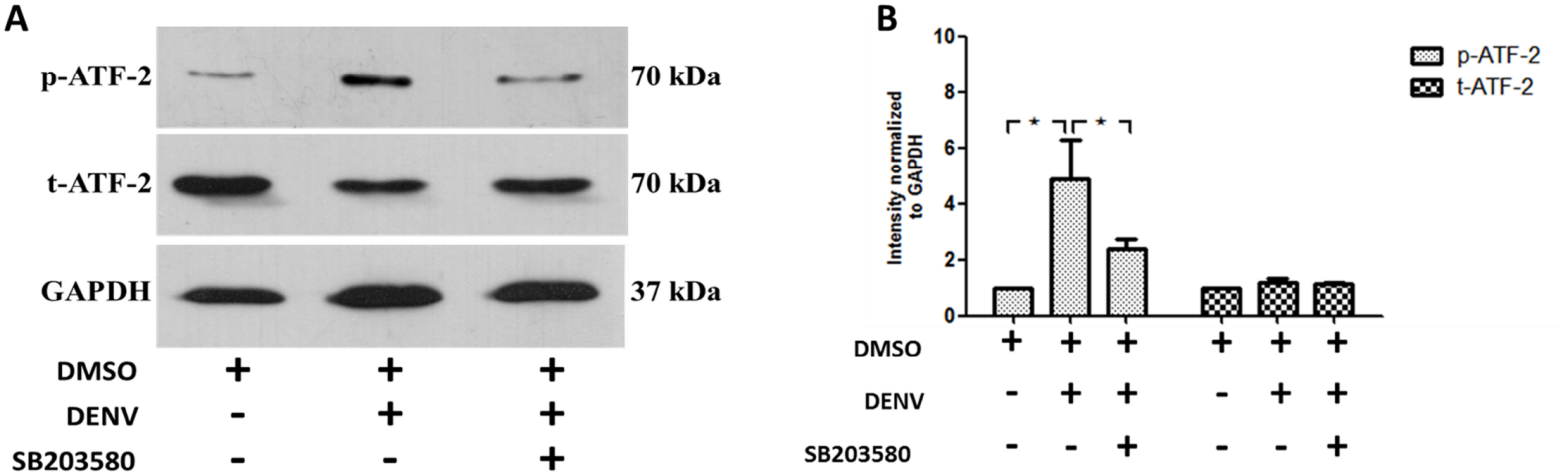
SB203580 treatment reduces the phosphorylation of ATF-2. Proteins were extracted from the liver tissue samples of 2%DMSO-treated (uninfected), 2%DMSO-treated DENV-infected, and SB203580-treated DENV-infected groups of mice. An additional cocktail of phosphatase inhibitors was added for maintaining the phosphorylated proteins and allowed them to standard Western blot analysis with specific antibodies. The result is shown (A) phosphorylated ATF-2 and total ATF-2 normalized to GAPDH. The results shown are representative of three independent experiments with three mice (n = 3) from each group. A densitometry analysis is conducted using the ImageJ software and is shown in (B).

We further explored the other downstream arm of p38 MAPK signaling, by investigating the role of ATF2, the downstream signaling molecules to p38 MAPK in DENV infection. Proteins were prepared from un-infected 2%DMSO treated, 2%DMSO-treated DENV-infected and SB203580-treated DENV-infected mice. Western blot analysis with antibodies directed against phosphorylated ATF2 and total ATF2 was conducted and normalized to GAPDH. Our results explains that DENV infection induced phosphorylation of ATF2 (Fig 13A) and SB203580 treatment in DENV-infected mice reduced expression of phosphorylated ATF2. The total ATF2 remains equal in each group of mice (Fig 13A). Densitometry analysis was also conducted and shown in Fig 13B.

**Fig 14:**
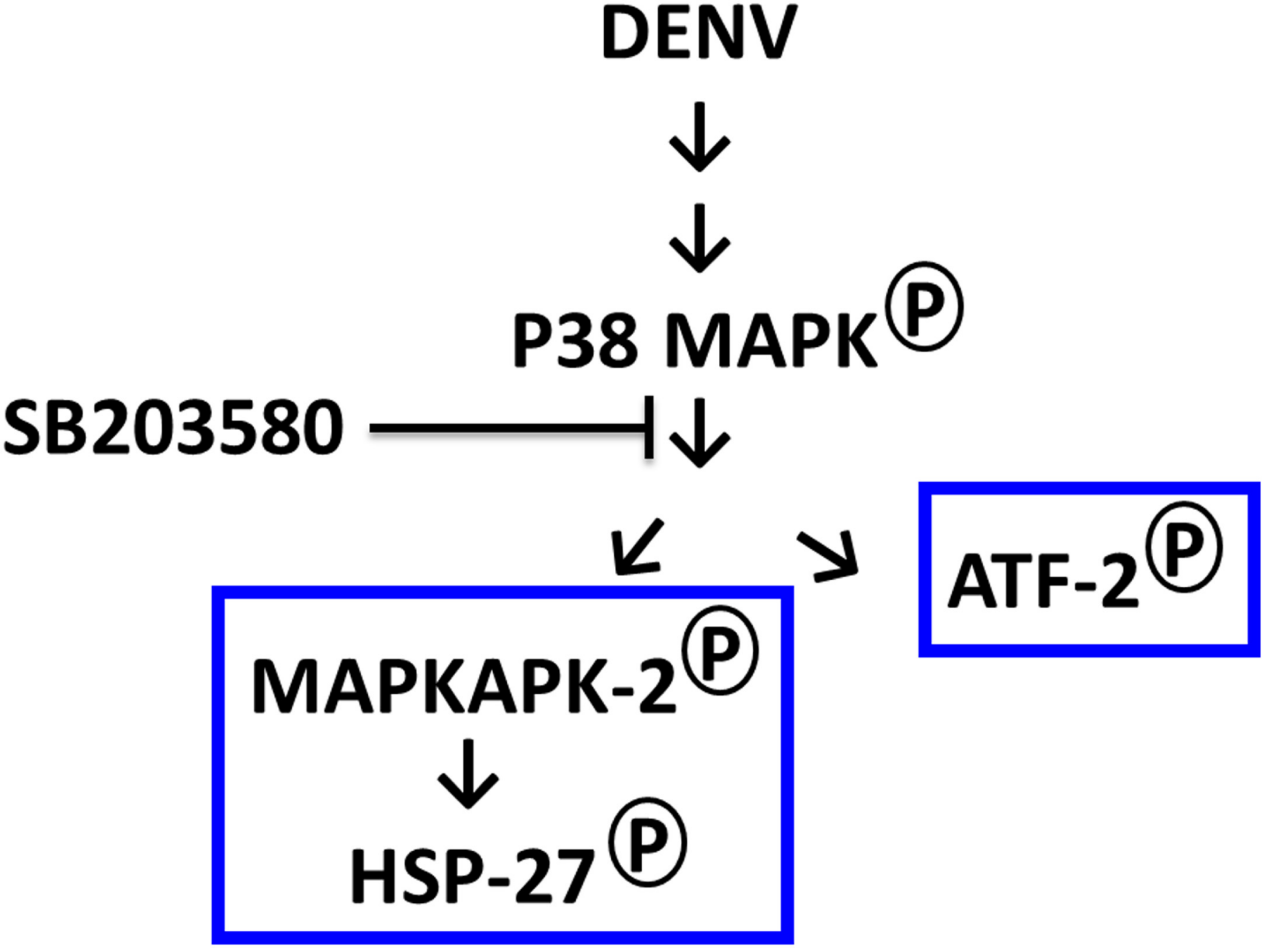
SB203580 does not directly reduce the phosphorylation of p38 MAPK but actually reduces both MAPKAPK2/HSP27 and ATF-2 arms of p38 MAPK signaling in DENV-infected mice.

We report here that DENV infection induces the phosphorylation of p38 MAPK, and the downstream signaling molecules to p38 MAPK including MAPKAPK2, HSP-27 and ATF-2 in DENV-infected mice (Fig 14). SB203580 didn’t directly reduce the phosphorylation of p38 MAPK but actually modulates both MAPKAPK2/HSP27 and ATF-2 arms of p38 MAPK signaling. Therefore, the modulation of downstream signaling to p38 MAPK by SB203580 reduces the DENV-induced liver injury.

## Discussion

Balb/c mice are susceptible to DENV infection and offer a convenient *in vivo* model of it [11–13]. An intravenous injection of DENV was used to study liver injury in Balb/c mice [11–13] because it produces prominent symptoms compared to DENV-infected mice infected by any other route [14, 48]. Viral particles were detectable in the liver and the classical markers of liver injury seen in patients were confirmed, including the elevation of liver transaminases and the characteristic histopathological changes. The WBC and platelet counts of the DENV-infected mice were reduced, suggesting leucopenia and thrombocytopenia, respectively, which are commonly seen in patients infected with DENV. These results are consistent with other models of DENV infection [63–65] and are similar to the symptoms in C57BL/6 mice [66] and human patients [67]. Although SB203580 improves the hematological profile, the levels of leucopenia and thrombocytopenia in the DENV-infected mice are still decreased relative to those in uninfected mice, similar to the results obtained with the chemical inhibitor of ERK1/2, FR180204 [48]. Therefore, SB203580 may be another option that can control the clinical parameters of DENV infection from the MAPK inhibitor family, but this requires confirmation by testing the additive or synergistic effects.

Elevated liver transaminases (ALT and AST) are used as clinical markers of the classical liver injury that occurs in mice infected with DENV, and are reported to be significantly elevated on day 7 after infection [11, 14, 68]. ALT and AST are also elevated in humans infected with DENV [69, 70]. No difference in ALT was seen with the addition of SB203580, though AST levels were altered in this study. It may be due to AST is more specific for liver inflammation than ALT, because the amount of AST in the liver is very much higher than that of ALT [8, 70, 71]. Elevated serum level of liver enzyme, gamma-glutamyl transpeptidase (GGT) was also correlated well with histology to study liver damage in alcoholism [72]. Similarly, in a mouse model of drug-induced chronic hepatitis GGT is significantly elevated [73]. A patient with DENV-induced fulminant hepatitis show increased GGT level [74]. The GGT expression in the livers of DENV-infected mice in our study is consistent with the above findings. A histological analysis of the liver tissues from DMSO-treated DENV-infected mice shows the classical signs of liver injury seen in DENV-infected patients [13, 75, 76]. These conditions were alleviated by treatment with SB203580, suggesting the efficacy of SB203580 in controlling the liver injury induced by DENV infection.

We explored the molecular mechanism by which SB203580 controls this liver injury with a commercially available Mouse Apoptosis RT^2^ Profiler^™^ PCR Array System (Qiagen). The increased pro-inflammatory cytokine expression induced by DENV is consistent with various other studies. The expression of TNF-α family increases in patients with severe DENV disease [24, 25, 77–80]. *In vitro* hepatic cell lines also show similar results for TRAIL [19, 81]. In DENV-infected HepG2 cells, TNF-α is increased and the inhibition of p38MAPK reduces TNF-α expression [20]. Interestingly, in the present study, *Cd40L* and *Cd40* mRNA expression is also up-regulated in the DENV-infected mice. An important TNF receptor family member, CD40, and its ligand, Cd40L, are important modulators of the antiviral immune responses [82–85]. In DENV-infected dendritic cells, CD40–CD40L signaling underlies the immune-mediated responses [86]. This finding requires further investigation primarily in hepatic cell line to determine the immuno-regulatory role of CD40–CD40L signaling in DENV-induced liver cell apoptosis. FAS/FASL pathway induces apoptosis in vascular endothelial cells [26]. FAS-mediated apoptosis is also induced DENV-infected HepG2 cells and the interaction of DENV capsid protein with DAXX is involved in this induction [27]. FAS-associated death domain (FADD) is also up-regulated during DENV infection, with concurrent increases of TNF-α and IL-10 expression [87]. Our results from mRNA expression profile suggest the involvement of FAS-mediated cell death in DENV-infected mice. The activation of the cytoplasmic apoptotic caspase was also observed in the present study. Increased cleaved caspase 3 expression is seen during DENV infection [88, 89] and explains the apoptosis and organ injury during DENV infection [83, 90–94]. We observed similar results of increased cleaved caspase 3 expression and SB203580 treatment restricted the cleaved caspase 3 expression, thereby decreasing the DENV -induced liver injury.

Very specifically, we questioned how the apoptotic signals were modulated by SB203580 treatment. Our results in DENV-infected mice confirmed increased expressions of caspase 8 and caspase 9, and we observed SB203580 treatment reduces the activation of both caspase 8 and caspase 9 in DENV-infected mice. In a previous *in vitro* study, DENV infection induced apoptosis through both intrinsic and extrinsic pathway, by the activation of caspase 8 and caspase 9 [95]. Our results were consistent in the liver tissues of DENV-infected mice and we show here that SB203580 is able to modulate apoptosis in both extrinsic and intrinsic pathways. Finally, we validated the previous findings, the apoptotic pathway by DENV infection in mice is in a caspase dependent manner and reports the efficacy of SB203580 in controlling the DENV induced apoptotic pathways.

Increased apoptosis is observed with increased pro-apoptotic cytokines including TNF-α, IL-6 and TRAIL [78]. In our study, mRNA expressions of cytokines including TNF-α, IL-6 and IL-10, and chemokines including RANTES and IP-10 are up-regulated in DENV-infected mice and SB203580 treatment reduces these expressions in DENV-induced liver injury. Our results are consistent with the inclined serum levels of IL-10 and IP-10 in patients with severe DENV infection and are associated with T cell apoptosis [96]. Children with DENV infection show increased levels of and IL-6, and IL-10 and correlates with the severity of disease progression [97], [98]. Increased IL-6 expression in the lung epithelial cells by DENV infection was found to be NF-kB dependent and is associated with RANTES expression [99]. Increased expression of RANTES in DENV-infected mice show liver damage, with leukocyte activation and increased IL-6 expression [66]. Increased expression of RANTES is observed in intra-cerebral inoculation of DENV, which induces behavioral changes and encephalitis in C57BL/6 mice. Both RANTES and IP-10 were shown to be induced in human lung epithelial carcinoma cells (A549) infected with DENV [100]. Our research group also found similar results in DENV-infected HEK 293 cells and reported this is by NF-kB activation [101]. However, further information on these in *in vivo* models of DENV infection is still required. Interestingly, the efficacy of SB203580 in controlling the expression of other important cytokines involved in DENV infection also requires further investigations.

Increased apoptosis is also observed with increased cytokine expression of TNF-α, which contribute to increased phosphorylation of p38 MAPK in DENV infected HepG2 cells [20]. In DENV-infected HepG2 cells, SB203580 reduces the expression of RIPK2 and DENV-induced apoptosis [17]. Recently, SB203580 has been shown to improve the clinical manifestations and systemic inflammation in an animal model of DENV infection. This interesting study shows oral administration of SB203580 in immuno-competent AG129 mice, decreases the circulation of pro-inflammatory cytokines (TNF-α, IL-6 and MMP-9) and reduces the leakage resulting a better survival rate [54]. But interestingly, there is neither *in vitro* nor *in vivo* study which shows the mechanism by which SB203580 treatment modulates apoptosis in DENV infection.

To provide insight into this, we investigated whether DENV infection influences the phosphorylation of p38 MAPK in DENV-induced liver injury. We used the immuno-competent Balb/c mouse to understand the host immune responses, especially apoptosis in contribution to liver injury. Our results demonstrate that DENV infection induces the phosphorylation of p38 MAPK in mice and SB203580 treatment did not control the phosphorylation of p38 MAPK in DENV infected mice. This suggests SB203580 does not directly inhibit the phosphorylation of p38 MAPK. Similar results were observed in a cell-based system, treatment with TNF-α shows increased phosphorylation of p38 MAPK signaling, but treatment with SB203580 does not reduce the phosphorylation of p38 MAPK, but interestingly inhibits its downstream kinases, including MAPKAPK2 and ATF2 at both arms of p38 MAPK signaling [102].

We examined the same path, whether SB203580 reduces the phosphorylation of its downstream kinases including MAPKAPK2 and ATF-2 in DENV infection. We found that SB203580 treatment reduces the phosphorylation of both MAPKAPK2 and ATF-2 in DENV-induced liver injury. MAPKAPK2, is one of the direct downstream target of p38 MAPK signaling and its deficiency is reported as an important controlling factor in protecting brain from ischemic injury in mice [83]. The inhibition of MAPKAPK2 is also reported to block the p38 MAPK signaling on reperfusion, thereby reducing redox stress and apoptotic cell death [103]. Our results were consistent with these observations that DENV also induces p38 MAPK phosphorylation and we confirmed the ability of SB203580 to decrease the phosphorylation of MAPKAPK2 in DENV-induced liver injury. At the other arm of p38 MAPK signaling, ATF-2 is reported to be activated by signals from stress-activated protein kinases, including JNK and p38 MAPK [104]. In varicella-zoster virus infections, both p38 MAPK and JNK are activated, thereby activating of the downstream signal ATF2 [105]. In an animal model of *Human immunodeficiency virus* infection, ATF2 phosphorylation is also inhibited by SB203580 treatment without inhibiting the phosphorylation of p38 MAPK [106]. Our results explains in DENV-infected mice, where SB203580 treatment reduces the phosphorylation of ATF-2 at another arm of p38 MAPK pathway, which give us more clues for decreased liver injury observed in this study.

HSP27 is a member in the family of small heat shock proteins that act in various cellular responses, including apoptosis [107]. The activation of MAPKAPK2 is also reported to increase vascular permeability, together with activated HSP27, the direct downstream signal to MAPKAPK2 and SB203580 is reported to be effective in controlling vascular permeability [108]. In another study of *respiratory syncytial virus* (RSV) infection, both p38 MAPK and HSP27 are required for increased human epithelial membrane permeability, and SB203580 controls the infectivity of RSV by attenuating this membrane permeability [109]. We also found increased phosphorylation of HSP27 in DENV-infected mice, which may directly come from the p38 MAPK and MAPKAPK2 phosphorylation signaling. SB203580 treatment decreases the phosphorylation of HSP27 by the inhibition of MAPKAPK2. As DENV infection also contributes to vascular permeability [110–113], this aspect needs further investigation.

Our results confirm that p38 MAPK and its downstream targets plays important roles in DENV-induced liver injury. DENV induces apoptosis by the induced phosphorylation of MAPKAPK2, HSP27 and ATF-2 and SB203580 treatment reduces the DENV-induced apoptosis, by inhibiting the phosphorylation of MAPKAPK2, HSP27 and ATF2. Since the inhibitor did not reduce p38 MAPK phosphorylation, there is a possibility that SB203580 may be acting directly on downstream molecules as well as p38 MAPK. Our study supports the previous studies, as p38 MAPK plays an important role in the induction of pro-inflammatory cytokine TNF-α during DENV infection [20] and modulation of inflammation and pathology in DENV infected mice by p38 MAPK inhibitor, SB203580 [54]. However, we open up the path to further investigate the downstream signals towards MAPK pathway, which is very important to better understand the pathogenesis of DENV infection.

## Conclusion

The p38 MAPK inhibitor, SB203580 treatment in DENV-infected mice reverses the AST, GGT and histopathology of the liver and controls the cytokine and chemokine responses. The apoptosis signaling in DENV-infected mice via both intrinsic and extrinsic pathway is modulated by SB203580, by reducing the phosphorylation of downstream signaling molecules to p38 MAPK including MAPKAPK2, HSP27 and ATF-2.

## Supporting Information

S1 Table SB203580 treatment modulates the apoptotic gene expression profile in DENV-infected mice. To explore the molecular mechanism by which SB203580 reduces liver damage, screening experiments were conducted with a commercially available Mouse Apoptosis RT^2^ Profiler^™^ PCR Array System (Qiagen). The full list of gene expression profile of the apoptosis related genes in DENV-infected mice and the effect of SB203580 treatment to those genes were shown in the S1 Table. The results were normalized to un-infected 2%DMSO-treated mouse. Actin is used as the housekeeping gene for normalizing the expression profile.
(PDF)
