# Supplementary material for: SB203580 Modulates p38 MAPK Signaling and Dengue Virus-Induced Liver Injury by Reducing MAPKAPK2, HSP27, and ATF2 Phosphorylation
Source: PLoS One. 2016 Feb 22;11(2):e0149486. doi: 10.1371/journal.pone.0149486 (PMC4764010; doi:10.1371/journal.pone.0149486)
Supplement: S1 Table — To explore the molecular mechanism by which SB203580 reduces liver damage, screening experiments were conducted with a commercially available Mouse Apoptosis RT2 Profiler™ PCR Array System (Qiagen). The full list of gene expression profile of the apoptosis related genes in DENV-infected mice and the effect of SB203580 treatment to those genes were shown in the S1 Table. The results were normalized to un-infected 2%DMSO-treated mouse. Actin is used as the housekeeping gene for normalizing the expression profile. (PDF) [file pone.0149486.s001.pdf]

S1 Table: Apoptotic gene expression profiles of DENV-infected DMSO-treated mice and DENV-infected SB203580 treated mice (Normalized with those of uninfected DMSO-treated mice)

Gene	Gene Description	DMSO+DENV	DENV+SB203580
Abl1	C-abl oncogene 1, non-receptor tyrosine kinase	2.5315	1.4538
Aifm1	Apoptosis-inducing factor, mitochondrion-associated 1	1.8790	1.9589
Akt1	Thymoma viral proto-oncogene 1	1.9862	2.2815
Anxa5	Annexin A5	1.3013	1.6020
Apaf1	Apoptotic peptidase activating factor 1	2.5669	1.8405
Api5	Apoptosis inhibitor 5	0.4540	0.8735
Atf5	Activating transcription factor 5	1.1728	1.3472
Bad	BCL2-associated agonist of cell death	1.9725	1.1407
Bag1	Bcl2-associated athanogene 1	1.9453	1.7290
Bag3	Bcl2-associated athanogene 3	1.1647	2.5491
Bak1	BCL2-antagonist/killer 1	0.9794	1.2311
Bax	Bcl2-associated X protein	3.3173	1.1730
Bcl10	B-cell leukemia/lymphoma 10	1.8921	1.3286
Bcl2	B-cell leukemia/lymphoma 2	0.4105	0.6701
Bcl2a1a	B-cell leukemia/lymphoma 2 related protein A1a	11.3924	6.4082
Bcl2l1	Bcl2-like 1	0.9231	0.7870
Bcl2l10	Bcl2-like 10	1.1173	0.0040
Bcl2l11	BCL2-like 11 (apoptosis facilitator)	0.9794	1.0425
Bcl2l2	Bcl2-like 2	1.8025	1.1487
Bid	BH3 interacting domain death agonist	2.3134	1.8660
Birc2	Baculoviral IAP repeat-containing 2	3.2266	2.4967
Birc3	Baculoviral IAP repeat-containing 3	1.6021	1.6702
Birc5	Baculoviral IAP repeat-containing 5	0.6643	0.9329
Bnip2	BCL2/adenovirus E1B interacting protein 2	0.9931	1.1975
Bnip3	BCL2/adenovirus E1B interacting protein 3	2.4453	0.9331
Bnip3l	BCL2/adenovirus E1B interacting protein 3-like	1.6586	1.4141
Bok	BCL2-related ovarian killer protein	1.3755	1.0942
Card10	Caspase recruitment domain family, member 10	1.0718	1.2835
Casp1	Caspase 1	4.1411	5.4642

Casp12	Caspase 12	4.7899	2.1737
Casp14	Caspase 14	2.5491	1.1407
Casp2	Caspase 2	2.4284	1.8531
Casp3	Caspase 3	2.1886	1.8277
Casp4	Caspase 4, apoptosis-related cysteine peptidase	1.9053	1.3288
Casp6	Caspase 6	1.8661	2.0279
Casp7	Caspase 7	1.6021	0.8645
Casp8	Caspase 8	2.1140	1.9588
Casp9	Caspase 9	1.9588	1.1647
Cd40	CD40 antigen	3.0314	1.3850
Cd40lg	CD40 ligand	1.7411	0.5323
Cd70	CD70 antigen	35.1268	11.7078
Cflar	CASP8 and FADD-like apoptosis regulator	1.6935	1.2923
Cidea	Cell death-inducing DNA fragmentation factor, alpha subunit-like effector A	8.3977	2.3295
Cideb	Cell death-inducing DNA fragmentation factor, alpha subunit-like effector B	2.2191	2.0562
Cradd	CASP2 and RIPK1 domain containing adaptor with death domain	4.6268	2.4286
Dad1	Defender against cell death 1	0.8652	1.8186
Dapk1	Death associated protein kinase 1	0.9862	0.7422
Dffa	DNA fragmentation factor, alpha subunit	1.5369	1.2924
Dffb	DNA fragmentation factor, beta subunit	3.4105	1.6585
Diablo	Diablo homolog (Drosophila)	1.2397	0.9330
Fadd	Fas (TNFRSF6)-associated via death domain	2.4623	2.0705
Fas	Fas (TNF receptor superfamily member 6)	1.4948	0.7422
Fasl	Fas ligand (TNF superfamily, member 6)	11.9588	7.8904
Gadd45a	Growth arrest and DNA-damage-inducible 45 alpha	2.4284	0.6112
Igf1r	Insulin-like growth factor I receptor	1.7777	1.6022
Il10	Interleukin 10	10.1261	1.0278
Lhx4	LIM homeobox protein 4	0.0179	0.0080
Ltbr	Lymphotoxin B receptor	2.0000	1.0210
Mapk1	Mitogen-activated protein kinase 1	1.3660	1.0867
Mcl1	Myeloid cell leukemia sequence 1	2.0000	1.3756
Naip1	NLR family, apoptosis inhibitory protein 1	0.8970	1.3518
Naip2	NLR family, apoptosis inhibitory protein 2	0.4743	0.6824
Nfkb1	Nuclear factor of kappa light polypeptide gene enhancer in B-cells 1, p105	1.7053	1.4439

Nme5	Non-metastatic cells 5, protein expressed in (nucleoside-diphosphate kinase)	3.6553	3.2488
Nod1	Nucleotide-binding oligomerization domain containing 1	2.0420	1.1727
Nol3	Nucleolar protein 3 (apoptosis repressor with CARD domain)	0.2501	0.2666
Polb	Polymerase (DNA directed), beta	1.1975	1.3473
Prdx2	Peroxiredoxin 2	1.0943	1.1408
Pycard	PYD and CARD domain containing	3.3870	1.7413
Ripk1	Receptor (TNFRSF)-interacting serine-threonine kinase 1	0.8409	0.7900
Tnf	Tumor necrosis factor	2.5491	1.1407
Tnfrsf10b	Tumor necrosis factor receptor superfamily, member 10b	3.1167	4.1125
Tnfrsf11b	Tumor necrosis factor receptor superfamily, member 11b (osteoprotegerin)	2.0849	1.6246
Tnfrsf1a	Tumor necrosis factor receptor superfamily, member 1a	2.1735	1.4241
Tnfsf10	Tumor necrosis factor (ligand) superfamily, member 10	11.3924	3.8370
Tnfsf12	Tumor necrosis factor (ligand) superfamily, member 12	2.1585	1.4045
Traf1	Tnf receptor-associated factor 1	2.9690	0.9332
Traf2	Tnf receptor-associated factor 2	1.4641	1.2312
Traf3	Tnf receptor-associated factor 3	0.7846	1.1892
Trp53	Transformation related protein 53	2.0849	1.6133
Trp53bp2	Transformation related protein 53 binding protein 2	1.6358	1.1330
Trp63	Transformation related protein 63	3.5801	1.1406
Trp73	Transformation related protein 73	2.5491	1.1407
Xiap	X-linked inhibitor of apoptosis	0.4698	0.6974
Actb	Actin, beta	1.0000	1.0000
